# Acquired somatic *TP53* or *PIK3CA* mutations are potential predictors of when polyps evolve into colorectal cancer

**DOI:** 10.18632/oncotarget.20376

**Published:** 2017-08-21

**Authors:** Pi-Yueh Chang, Jinn-Shiun Chen, Shih-Cheng Chang, Mei-Chia Wang, Nai-Chung Chang, Ying-Hao Wen, Wen-Sy Tsai, Wei-Hsiu Liu, Hsiu-Ling Liu, Jang-Jih Lu

**Affiliations:** ^1^ Department of Laboratory Medicine, Chang Gung Memorial Hospital at LinKou, Taoyuan, Taiwan; ^2^ Department of Medical Biotechnology and Laboratory Science, Chang Gung University, Taoyuan, Taiwan; ^3^ Department of Colorectal Surgery, Chang Gung Memorial Hospital at Linkou, Taoyuan, Taiwan

**Keywords:** next-generation sequencing, polyps, colorectal cancer, ampliseq cancer hotspot panel, synchronous neoplasm

## Abstract

Colorectal cancer (CRC) develops from accumulated mutations. However, which gene determines the malignant transformation from adenoma to carcinoma is still uncertain. Fifty-three formalin fixed paraffin-embedded polyps that had pathological findings from patients with hyperplasia, adenomatous, and tubular adenoma < 1 cm (non-neoplasia polyps, NNP, *n* = 27) or tubular adenoma ≥ 1 cm, tubulovillous and villous adenoma (neoplastic polyps, NP, *n* = 26) were recruited. Six paired synchronous polyps and cancer tissues and 50 independent fresh CRC tumors were also collected. All tissues were analyzed for their mutation genomes using next generation sequencing with a 50-gene panel. There were 40 types of somatic variants found in 7 genes, *APC* (43%), *KRAS* (28%), *TP53* (11%), *FBXW7* (8%), *GNAS* (4%), *SMAD4* (2%), and *BRAF* (2%), and they were detected in 32 (60%) polyps. If combined with the mutation spectrum found in CRC tissues, a significant increase in the mutation rate in *TP53* and *PIK3CA* from NNP, NP, early and late stage carcinoma (7%, 15%, 33.3% and 65% for TP53, *p* < 0.001; 0%, 0%, 23.3% and 25% for *PIK3CA*, *p* = 0.002) were noticed. Furthermore, distinct molecular features can be found in five pairs of synchronous polyps and tumors. However, *TP53* or *PIK3CA* mutations can be found in tumor tissues but not in polyps. By systematically investigating the genome from polyps to tumor tissues, we demonstrated that acquired *TP53* or *PIK3CA* somatic mutations are potential predictors for malignancy development. These results may aid in the identification of high risk individuals with tissues harboring mutations in these two genes.

## INTRODUCTION

The normal epithelial-adenoma-carcinoma axis was proposed thirty years ago by Vogelstein and Fearon [1]. The genetic changes involve the initial loss or mutation of *APC/β-catenin* genes for the formation of aberrant crypts foci, mutations of *KRAS/BRAF* to advanced adenoma, and other acquired mutations for transformation to carcinoma [2]. The tumorigenesis took several decades. Epidemiology studies have demonstrated an average 2% cancer transformation rate of polyps every year [3] that result in a 5∼6% lifetime probability of developing into colorectal cancer in an average-risk individual at 68 years of age [2]. The central theme of this theory is the necessity of unstable chromosomes by the loss of APC function in the first stage and the accumulation of sequentially acquired mutations to exert tumor characteristics in a time-dependent manner. However, in our previous study, *APC* was not the most frequently mutated gene in 50 colorectal (CRC) tumors, nor did it have the highest mutated allelic frequency within one tumor [4]. Instead, *TP53* gained the highest mutation rate (46%) among 20 frequently aberrant genes in CRC and almost had the highest alleice frequency if multiple gene mutations were found within an individual tumor. This observation challenged the dogma for the essential presence of an *APC* mutation and raised the possibility of multiple different mutation pathways that determine cancer transformation.

To elucidate the role of each gene during tumorigenesis, it is warranted to identify the mutation profiles of each stage of pre-cancerous neoplasia. Recently, Sievers *et al*. [5] surveyed the genetic landscapes of 48 small (< 9 mm) resected polyps from 36 asymptomatic patients after a follow-up by routine CT scan for 1–3 years. They concluded that there was no significant correlation between the genetic profile and the adenoma growth rate. However, they found multiple subclones with lower allelic frequency of driver mutations coexisted early in small polyps and might cause malignancy transformation till it acquired additional driver mutations or received non-genetic changes. The results imply the importance of qualitative and quantitative measurement of tumor genome. In this study, we conduct a retrospective cohort study to investigate the mutation patterns of 53 archived polyp tissues with histologically proven findings from patients with hyperplasia to villous adenoma by performing next generation sequencing (NGS) with a 50-gene panel. The platform is the same as the one we used in our previous study for 50 CRC fresh tumor tissues. Therefore, we can parallel the data from the two studies to observe the changes in the altered somatic gene pattern from the initiation of cell proliferation, the pre-cancerous stage, to the early and late stages of cancer in a cross-sectional view. This study may reveal how a polyp evolves into cancer through multiple steps of genome alteration and reestablish the proliferation tree map by quantitating the relative allelic frequency of each mutated gene within one tumor.

However, mutation patterns from cross-sectional studies are affected by individual host genomic background and unaccountable environmental factors of the study cohorts. The best way to investigate the contribution of each gene in cancer formation should be the longitudinal follow-up of a single polyp from a benign state to malignancy. As it is difficult to monitor the evolution of the cancer genome in that way, we collected six pairs of synchronous polyp and tumor tissues from 6 individual patients to focus on the differences in the gene spectrum of pre-cancer and cancer lesions within the same individual. All the information can help us elucidate the dominant gene and pathway paving the road to cancer.

## RESULTS

### General patient characteristics and germline variants in patients with polyps compared to those in CRC patients

**Figure 1:**
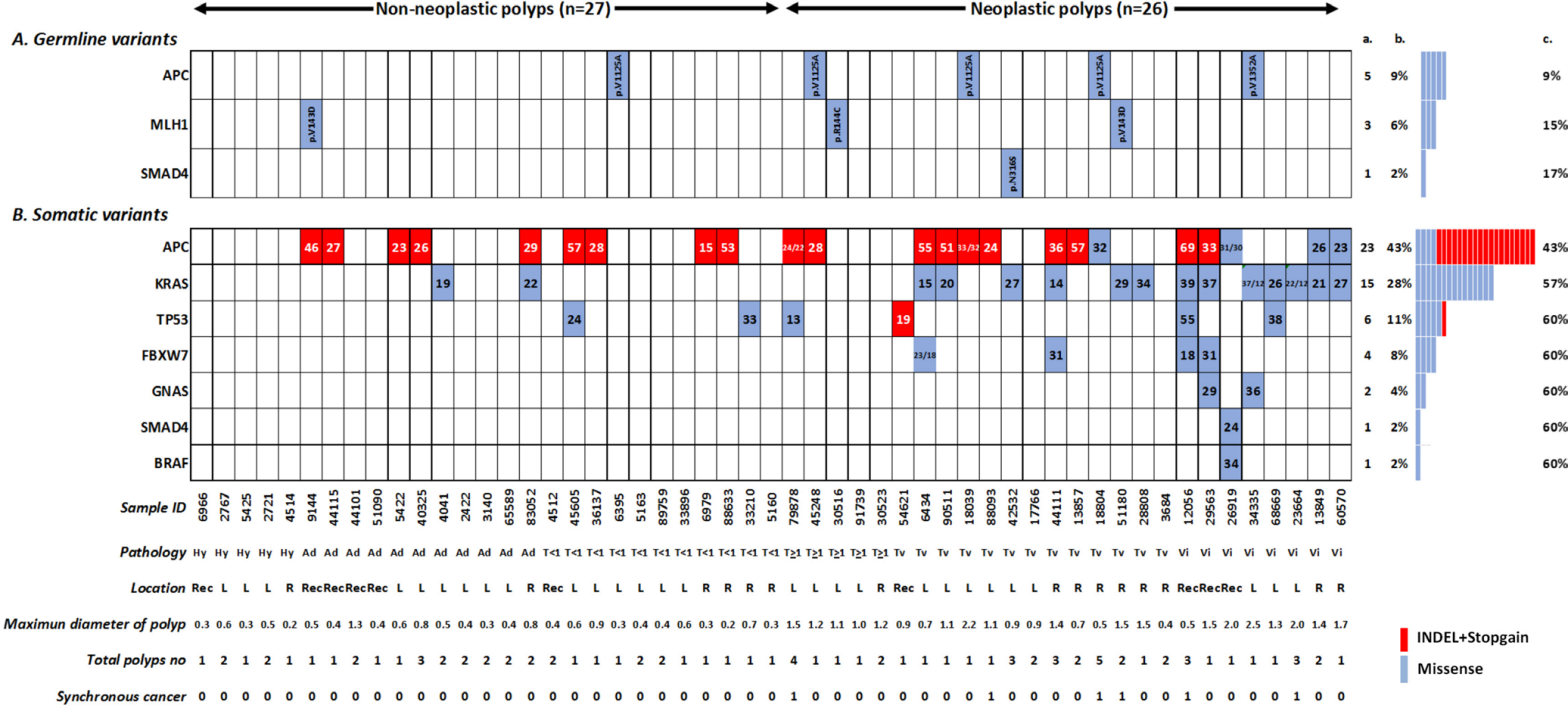
Distribution of 5 germline variants in 3 cancer-susceptible genes (**A**) and 40 somatic variants in 7 genes (**B**) in 53 polyp tissues. Sample order is lined up by pathology finding and lesion location. The color and number in the square cell represents the variant type (red denotes an indel or nonsense mutation, and blue denotes missense mutation) and variant allelic frequency. More than one number in one cell indicates multiple mutations in the same gene. Columns a and b denote the number and percentage of samples altered per gene; column c denotes the accumulated percentage of samples with mutated genes. The patient information denoted at the bottom of the figure includes the sample ID, histological classification, location, maximum diameters of the polyp (cm), number of polyps and whether synchronous cancer was present (1 stands for yes). “Hy” = Hyperplasia; “Ad” = adenomatous; “T < 1” = tubular size < 1 cm; “T ≥ 1” = tubular size ≥ 1 cm; “Tv” = tubulovillous; ”Vi” = villous; “Re” = rectum; “R” = right side; and “L” = left side.

Table 1 summarizes the clinical features of 53 patients with polyps. The number of patients with non-neoplastic polyps (NNP, *n* = 27) was equal to the number with neoplastic polyps (NP, *n* = 26). Of the enrolled participants, 45% had more than one polyp, and they were diagnosed at an average of 58 years old. Figure 1A shows nine (17%, 9/53) patients who were identified as carrying germline variants. Among them, two had diagnoses of NNP, and the remaining seven patients had advanced polyps. Table 2 summarizes the germline variants found in the polyps and CRC groups. In patients with polyps, 5 germline variants were detected in the three most common CRC-susceptible genes, *APC*, *MLH-1* and *SMAD4*. The tolerant *APC* variants V1125A and V1352A were represented as germline mutation hotspots (9%, 5/53). *MLH1* V143D and R144C were predicted as damaging mutations, and one rare *SMAD4* N316S variant was discovered in one patient. On the other hand, the damaging *CDH1* T340A, damaging *NRAS* G138R and tolerated *MLH-1* R148Q variant were exclusively present in the cancer group. Among the eight cancer-predisposing germline mutations, all were minor alleles in the Taiwanese except for the *MLH1* V143D mutation, which had a 6.0% frequency in the general Taiwanese population.

**Table 1:** Clinical characteristics of 53 patients with polyps

Clinical Features		Patient no (%)
Sex	Male	40 (75%)
	Female	13 (25%)
Age	Mean (range)	58 (26–75)
Pathology classification	**Non-neoplasia polyps**	27
	Hyperplasia	5 (9%)
	Adenomatous	11 (21%)
	Tubular adenoma < 1 cm	11 (21%)
	**Neoplastic polyps**	26
	Tubular adenoma ≥ 1 cm	5 (9%)
	Tubulovillous	13 (25%)
	Villous adenoma	8 (15%)
Patients with > 1 polyps (%)		24 (45%)
Patients with synchronous cancer (%)		6^$^ (11%)
Polyps location	Right*	15 (28%)
	Left**	28 (53%)
	Rectum	10 (19%)
Treatment	Polypectomy removed	53 (100%)

$: 6 patients provided tumor FFPE specimen for comparison of mutation patterns with those in paired polyps.

*Right side including polyps located in cecum, ascending, transverse and hepatic flexure.

**Left side including polyps located in sigmoid, descending and splenic flexure.

**Table 2:** Germline mutation list and frequency comparison found in 53 polyps patients and 50 colorectal cancer patients

Gene	Locus	Exon/CDS change	A. A. change	SIFT prediction	% in polyps	% in CRC*	Allelic frequency in Taiwanese**
***APC***	chr5:112174665	Exon15/c.T3374C	p.V1125A	Tolerated	7.5%(4/53)	2% (1/50)	0.7%
	chr5:112175346	Exon15/c.T4055C	p.V1352A	Tolerated	1.9% (1/53)	2% (1/50)	0.0%
***MLH1***	chr3:37067240	Exon11/c.T428A	p.V143D	**Damaging**	3.8% (2/53)	0% (0/50)	6.0%
	chr3:37067242	Exon11/c.C430T	p.R144C	**Damaging**	1.9% (1/53)	0% (0/50)	1.3%
	chr3:37067255	Exon11/c.G443A	p.R148Q	Tolerated	0.0% (0/53)	2% (1/50)	0.0%
***SMAD4***	chr18:48586278	Exon8/c.A947G	p.N316S	Tolerated	1.9% (1/53)	0% (0/50)	0.0%
***CDH1***	chr16:68846047	Exon8/c.A1018G	p.T340A	**Damaging**	0.0% (0/53)	6% (3/50)	0.7%
***NRAS***	chr1:115252228	Exon4/c.G412A	p.G138R	**Damaging**	0.0% (0/53)	2% (1/50)	0.0%
**Total**					17% (9/53)	12% (6/50)	

*Data have been published in previous study (P-Y Chang *et al*., Oncotarget, 2016).

**Data were collected from 150 healthy Taiwanese people with age larger than 70 years old by whole genome sequencing.

### Somatic variants in patients with polyps

After the subtraction of inherited variants, somatic mutations in each FFPE polyp sample were identified. *APC* (43%), followed by *KRAS* (28%) and *TP53* (11%), are the most frequently mutated genes and those three genes cover 60% of the polyp patients (Figure 1B). In sum, a total of 40 somatic variants in 7 genes in the polyps group were identified and confirmed by Sanger sequencing (variant information was listed in Table 3). Regarding the variant effect, 20 missense mutations (50%), 4 indel mutations (10%), and 16 nonsense mutations (40%) were identified. Among these, 3 (7.5%) variants, *APC* S1344*, *APC* F1500fs and *GNAS* R186C, were novel and were not reported in the COSMIC database. Missense mutations of in codons 12 and 13 of *KRAS* (including G12V, G12D, and G13D) were the most frequently observed variants in the samples (23%, 12/53). Indel and nonsense mutations, which can lead to truncated proteins, were distributed mostly in the *APC* and *TP53* genes.

**Table 3:** Forty somatic variants distributed on 7 genes in 53 polyps tissues

Gene name (RefSeq ID)	Nucleotide Change	Amino Acid Change	Variant effect	Patient no
TP53 (NM_000546)	c.C321A	COSM45040_p.Y107*	stopgain	1
	c.A641G	COSM43687_p.H214R	nonsynonymous missense	1
	c.A659G	COSM10758_p.Y220C	nonsynonymous missense	1
	c.A701G	COSM10725_p.Y234C	nonsynonymous missense	1
	c.G731A	COSM10883_p.G244D	nonsynonymous missense	1
	c.G733A	COSM6932_p.G245S	nonsynonymous missense	1
KRAS (NM_033360)	c.G34A	COSM517_p.G12S	nonsynonymous missense	1
	c.G34T	COSM516_p.G12C	nonsynonymous missense	1
	c.G35A	COSM521_p.G12D	nonsynonymous missense	5
	c.G35T	COSM520_p.G12V	nonsynonymous missense	3
	c.G38A	COSM532_p.G13D	nonsynonymous missense	4
	c.A183C	COSM554_p.Q61H	nonsynonymous missense	2
	c.G436A	COSM19404_p.A146T	nonsynonymous missense	1
APC (NM_000038)	c.C2626T	COSM18852_p.R876*	stopgain	2
	c.C3340T	COSM13125_p.R1114*	stopgain	1
	c.C3367T	COSM1432255_p.Q1123*	stopgain	1
	c.G3856T	COSM18772_p.E1286*	stopgain	2
	c.C3871T	COSM19072_p.Q1291*	stopgain	2
	c.C3880T	COSM18960_p.Q1294*	stopgain	1
	c.3894_3895delTG	COSM18980 p.S1298fs	frameshift	1
	c.C3907T	COSM13728_p.Q1303*	stopgain	1
	c.G3916T	COSM18760_p.E1306*	stopgain	2
	c.C4031G	p.S1344*	stopgain	1
	c.G4033T	COSM18759_p.E1345*	stopgain	1
	c.4060delT	COSM292626_p.S1355fs*60	frameshift	1
	c.C4067G	COSM18779_p.S1356*	stopgain	1
	c.G4120T	COSM19085_p.E1374*	stopgain	1
	c.C4285T	COSM18836_p.Q1429*	stopgain	1
	c.C4348T	COSM13127_p.R1450*	stopgain	4
	c.4385_4388delAGAG	COSM1432412_p.E1464fs*8	frameshift	1
	c.4498delT	p.F1500fs	frameshift	1
	c.G4660T	COSM33818_p.E1554*	stopgain	1
SMAD4 (NM_005359)	c.G1082A	COSM14122_p.R361H	nonsynonymous missense	1
FBXW7 (NM_018315)	c.C1153T	COSM170725_p.R385C	nonsynonymous missense	1
	c.G1196A	COSM94297_p.R399Q	nonsynonymous missense	1
	c.G1196T	COSM99619_p.R399L	nonsynonymous missense	1
	c.C1273T	COSM74637_p.R425C	nonsynonymous missense	1
	c.C1505T	COSM295016_p.S502L	nonsynonymous missense	1
BRAF (NM_004333)	c.T1799A	COSM476_p.V600E	nonsynonymous missense	1
GNAS (NM_001077489)	c.C556T	p.R186C	nonsynonymous missense	2

Two novel variants on *APC* gene and one variants on *GNAS* gene which are not recorded in the COSMIC database are shaded. Nomenclature of variants follows the guideline of Human Genome Variation Society. *stands for stop codon.

### Gradually acquired somatic mutations in polyps and CRC tissue samples and their correlation with clinicopathological factors

For better clarification of the molecular features alone with the development from adenoma-to-cancer with various clinicopathological factors, we enrolled our previous study results for cross-sectional comparison of the genetic alteration (somatic mutation profiles of 50 CRC patients are in Supplementary Figure 1). Four subgroups were stratified: NNP, NP, CRC early stage and CRC late stage.

**Figure 2:**
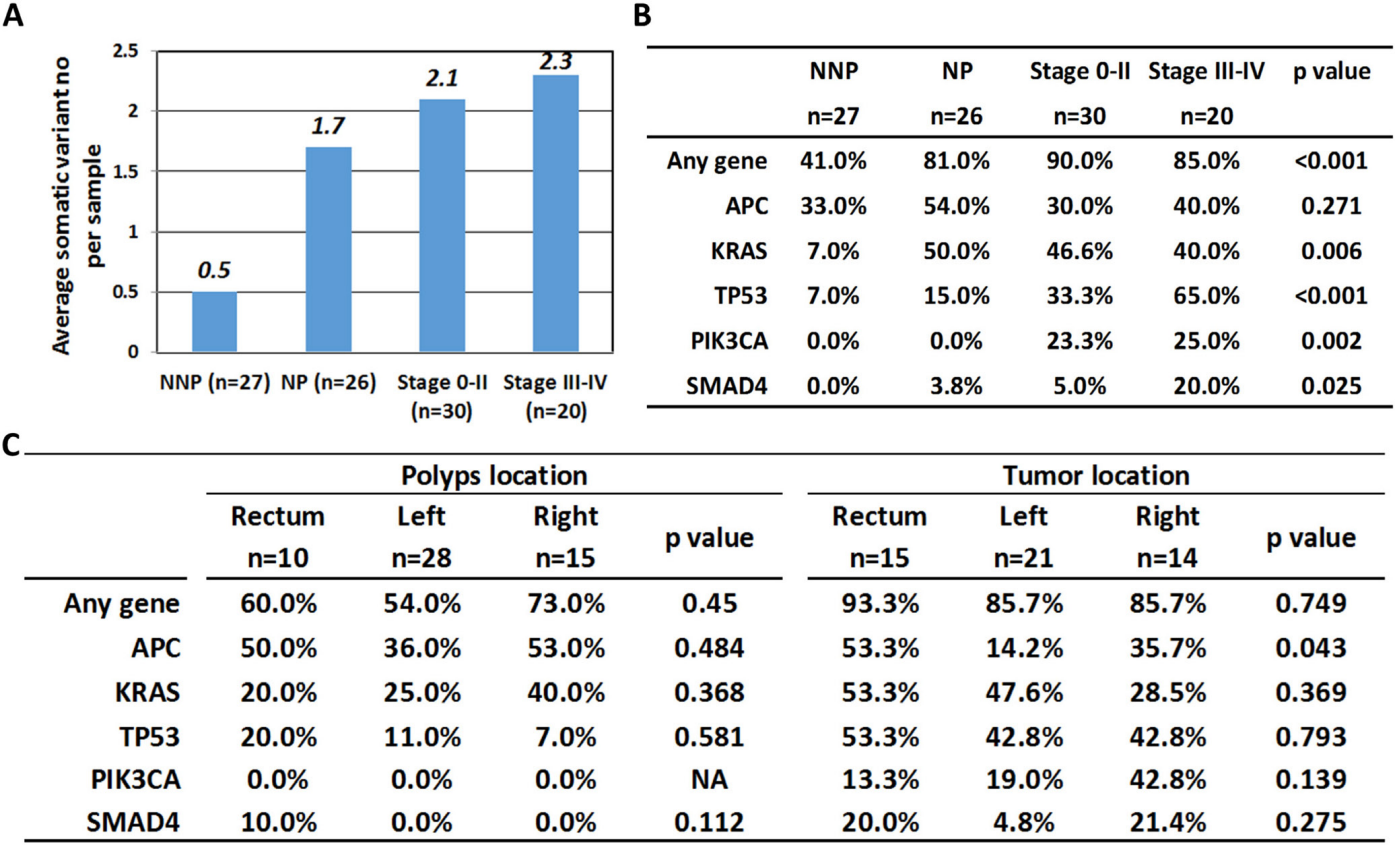
Correlation of gene mutation number and mutation rate with clinicopathological factors One hundred three samples were divided into 4 categories. NNP: non-neoplastic polyps, NP: neoplastic polyps, stage 0–II: colorectal cancer stage 0–II and stage III–IV: CRC with stage III–IV. (**A**) Comparison of average somatic variant number in 4 subgroups. (**B**) Comparison of mutation rate of top five mutated genes. (**C**) Correlation of mutation rate of top five mutated genes with lesion location in polyps and CRC groups.

First, the average numbers of somatic variants in the four subgroups were 0.5, 1.7, 2.1 and 2.3 (Figure 2A). Figure 2B summarized the mutation rate of the top 5 mutated genes in different stages of neoplasm. It was clear that with the exception of *APC* with the same high mutation rate from the initiation stage to the end stage of cancer, the *KRAS, TP53, PIK3CA* and *SMAD4* gene mutation rates were elevated when malignancy developed (*p < 0.05*), and a significant increase of the positive rate on *TP53* and *PIK3CA* from NNP, NP, early stage and late stage of carcinoma (7%, 15%, 33.3%, and 65% for *TP53, p < 0.001*; 0%, 0%, 23.3%, and 25% for *PIK3CA, p = 0.002*) were also observed. Figure 2C further demonstrated the individual gene mutation rate with the location. The statistical results indicated no particular tendency in the mutation pattern at different locations of the large intestine lumen.

Second, Supplementary Figure 2 illustrated the spatial distribution of the variants in the 5 most frequently mutated genes in the polyps and CRC tissues. In overview, variants in each gene were located in the well-known mutation areas regardless of whether they were derived from polyps or tumor tissues. For example, all mutations on the *APC* gene were distributed in the β-catenin-binding domain, and the variants in the *TP53* gene were located in the DNA-binding domain. *KRAS* mutations from adenomas and CRC occurred exclusively in codons 12, 13, 60, 61 and 146. Unexpectedly, no *PIK3CA* mutation was found in the 53 polyp tissues, in which mutations in Exon 9 and Exon 20 are frequently reported in CRC. The similar mutation locations in each gene in both groups indicates the continuity of the deleterious genome from adenoma to carcinoma.

### Distinct mutation profiles of synchronous polyps and tumors in one individual

**Figure 3:**
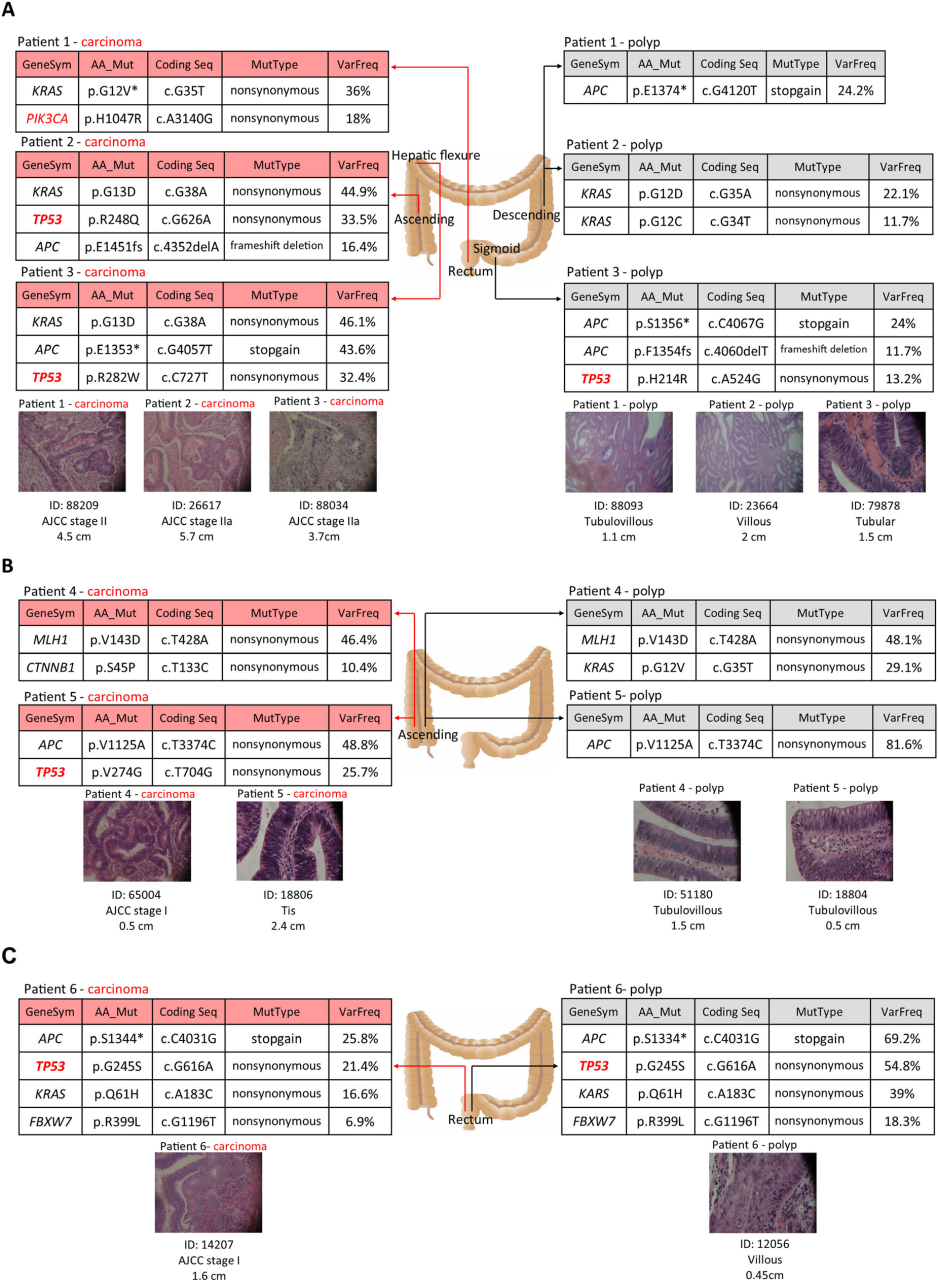
Distinct molecular features of synchronous polyps and cancer in six individuals (**A**) Mutation profiles found in three patients with polyps and cancer at different locations. (**B**) Mutation profiles in two patients with polyps and cancer at the same location and with germline mutations. (**C**) Mutation patterns in one patient with polyp and cancer at the same location. The histology of each lesion is shown by the hematoxylin and eosin stained slide with ×40 magnification. The mutation details of each neoplasm are summarized as a table.

Figure 3 demonstrates the comparison of the mutation patterns of paired polyp and carcinoma tissues in six patients. Figure 3A includes three patients (patient 1–3) whose polyps and cancer located at different sites within the large intestine. The results indicate that completely different mutation alleles could exist in distant lesions. For example, patient 1 has one tubulovillous polyp located in the descending intestine with an *APC* E1374* mutation. However, their stage II carcinoma located in the rectum presented *KRAS* G12V and *PIK3CA* H1047R, two different mutations. In patient 2, although *KRAS* mutations were found in both the polyp and carcinoma, the mutation alleles were G12D and G12C in the polyp and G13D in the carcinoma, which all have been confirmed by Sanger sequencing. The same finding was observed in patient 3. *APC* and *TP53* were mutated in both neoplasms with non-consistent mutation points. Figure 3B includes two patients with polyps and tumors that existed at the same ascending area. These two patients had germline mutations in *MLH1* and *APC* respectively. After excluding the germline mutation, distinct molecular features were also observed in paired lesions, even when they were located in the same area. Figure 3C demonstrates only one concordant pattern of two lesions at the same location within one patient in our study. In summary, independent mutation patterns were observed in synchronous lesions within one individual. However, the *TP53* and *PIK3CA* somatic mutations were found to be present in the majority of tumor tissues but not in the polyp lesions.

## DISCUSSION

The majority of colorectal cancers progress from polyps. In Taiwan, 25.8% of the average-risk population at age ≥ 50 years had polyps, and 22% of them were advanced neoplasms [6]. Synchronous polyps, which means two or more polyps developed simultaneously, are also prevalent in polyps population (∼25% [7, 8] and 45% in this study). According to the results of a large meta-analysis, individuals who performed polypectomy and found having synchronous polyps will have an odds ratio of 2.47 (95% CI, 1.74–3.50) to have a metachronous polyp in the future and an odds ratio of 5.15 (95% CI, 2.02–13.14) to have a metachronous CRC if the initial polyp was severe dysplasia [9]. This phenomenon echoes the observation that the causes of polyp production and progression to cancer are contributed to the genetic nature of cancer-susceptible individuals (20%) and environmental insults (80%) [10]. If individuals had the cancer-prone genome and were constantly under a cancer triggering environment, then they were at high risk to develop polyps or cancer repeatedly. Therefore, for cancer prevention purposes, we need a biomarker to identify individuals at high risk of having polyps (e.g. germline mutations), individuals with polyps at high risk to develop to cancer (e.g. specific somatic mutations).

Molecular alterations have been thoroughly investigated in CRC by high throughput sequencing platforms [11, 12]. However, few studies investigated the molecular background of adenomas and only screened in limited clinical specimen. Zhou *et al*. used the exome capture sequencing method to investigate the genetic alterations in only one pair of a tubular adenoma and adenocarcinoma that were 4 cm apart [13]. Kang *et al*. reported the intratumor heterogeneity by thoroughly measuring the point mutations, chromosome copy number and methylation in opposite sides of a 6 cm adenoma [14]. Another two studies focus the genome of adenomas specific in 36 patients with 48 small-sized polyps [5] and in 12 patients of familial adenomatous polyposis [15]. In the present study, we collected 53 polyps from 53 patients with pathology from non-neoplastic to neoplastic findings and combined the results from 50 CRC tissues in our previous study to comprehensively compare the germline and somatic mutation profiles by the same NGS cancer panel.

For germline mutations, our results reveal a 17% of mutation rate in cancer-susceptibility genes in the polyps group (Table 2). Most of the variants had significantly higher allelic frequencies than those in the general Taiwanese population and damaging effects on the protein function according to the SIFT prediction. *APC* is an autosomal dominant gene with 100% penetrance. Variants in the Wnt/β-Catenin pathway may contribute to colorectal cancer development [16]. In particular, the *APC* V1125A variant in this study has been reported previously in a Taiwanese CRC cohort study [17]. On the other hand, any kind of *MLH1* mutation carriers are reported to have an estimated 34% risk for males and 36% risk for females to develop into colorectal cancer at age 70 years [18]. In sum, the findings of this retrospective polyps study combined with the novel *CDH1* and *NRAS* germline mutations found in the CRC groups emphasize the existence of the Taiwanese-specific genome and provided candidate genes for cancer risk assessment in the general population.

For somatic mutations, the number of mutated genes and the mutation rates of specific genes significantly increased from non-neoplastic polyps to the late stage of carcinoma (Figure 2), which demonstrates that cancer might form when the acquired mutations reach the threshold. However, is the total number of mutated genes or the mutation of specific genes vital to cancer formation? By quantitative evaluation of the intra-tumor allelic frequency, we can anticipate the evolutionary history from adenoma to carcinoma. Although the allelic frequency is dependent on the percentage of tumor content in the original sample [12], the individual allelic frequency of multiple mutants occurring within one sample can still provide information about how subclones carrying different mutations evolved. In 53 polyp tissues, 15 samples had two or more somatic mutations, and nine of them had the highest mutant percentage in the *APC* gene. This phenomenon fits Volgestein's theory in which cells with an *APC* mutation at the initial stage acquired new mutations such as *KRAS* and *TP53* in the original clone when neoplasm grows (Supplementary Figure 3A). However, in the CRC group, the gene with the highest mutation frequency within one tumor is *TP53* which suggests that multiple alternative pathways other than *APC* mutations are involved [19]. Subclones that were not necessarily derived from the original *APC*-mutated clone may carry the essential driver mutations or defective pathways, such as *TP53* or *PIK3CA*, and gain a growth advantage with neoplasm progression (Supplementary Figure 3B).

The p53 protein induces G1 cell-cycle arrest and facilitates DNA repair prior to a cell committing to the process of DNA replication. The fact of p53 inactivation as a critical rate-limiting event can be evident by introducing a human *TP53* knock-in mouse model (Hupki mouse) [20]. Concordant with our finding, the elevated *TP53* mutation rate in CRC but not adenoma and its pathological role in CRC has been reported by other groups [21–23]. On the other hand, *PIK3CA* mutation is associated with phosphorylated AKT expression to decrease apoptosis and increase tumor invasion [24]. Activating mutations in *PIK3CA* are present in many cancer types, including colorectal cancer, with a 10–30% mutation rate [25]. However, *PIK3CA* mutation is uncommon in polyps. Vicki L.J. Whitehall *et al*. surveyed 426 colorectal polyps, and *PIK3CA* mutations were only present in 4 tubulovillous adenomas and 1 tubulovillous adenoma; no *PIK3CA* mutations were found in non-neoplastic polyps [26]. In our studies, 0% of *PIK3CA* mutations could be found in 53 polyp tissues, but 24% were found in the CRC group. More interestingly, *PIK3CA* and *TP53* mutations were mutually exclusive in CRC patients (Supplementary Figure 1) and contributed to a total of 68% of the mutation rate (34/50). This result demonstrated the crucial roles of these two genes in tumorigenesis.

For investigating the development of polyps and cancer under the same genetic background, the genomes of six pairs of synchronous polyps and tumors were compared. Independent molecular features and a right-sided tumor tendency of synchronous neoplasm within one individual were observed. Recent molecular pathological epidemiology (MPE) theory provokes a concept that the molecular features and behavior of tumor cells are influenced by host immunity and inflammation [27], as well as the interaction of various molecules [24, 28] and exposures to the local microbiome [29]. Concordant with our finding, Wheeler *et al*. recently reported on the distinct genomic profiling of 6 synchronous CRCs in one patient with Lynch syndrome. This result demonstrates that each lesion had a unique pattern of affected genes [30]. The continuous interaction of a growing neoplasm with the surrounding environment results in the tumor heterogeneity nature and the unpredictable development of any given lesion, especially when more than one polyp existed in one patient. However, we found that *TP53* or *PIK3CA* mutation always appeared in the carcinoma counterpart but not in the polyp site, except for one pair at a very adjacent location (patient 6 in Figure 3). The same finding has been reported by Haley’ s group [12]. They investigated the genomic profiles of 5 paired adenoma and invasive carcinoma tissues and found that *PIK3CA* mutations were only present in two of five carcinoma tissues and not in the adenoma. This result implies that the detection of the presence of *TP53* or *PIK3CA* mutations may serve as a predictor for being prone to the development of cancer.

In conclusion, this is the first comprehensive investigation of both polyps and the CRC genome using a next generation sequencing platform. The paired genome comparison of synchronous polyps and cancers also provides direct evidence of the impact of local environment and highlights the importance of acquiring and expanding subclones with *TP53* or *PIK3CA* mutations. The vital role of genetic alteration in the TP53 and PI3K pathway provides the possibility for designing a better screening strategy and targeted treatment to stop the tumorigenesis process before cancer transformation.

## MATERIALS AND METHODS

### Study patients

#### Polyp patients

Fifty-three participants who received polypectomy to remove polyps in a national cancer screening program at Chang Gung Memorial Hospital in Taiwan were enrolled between 2013 and 2015. Resected polyps were classified according to World Health Organization criteria and general consensus [31]. Histological findings were reported by experienced pathologists. The classification was divided into six categories: hyperplasia, adenomatous, tubular adenoma with largest diameter < 1 cm, tubular adenoma with largest diameter ≥ 1 cm, tubulovillous adenoma and villous adenoma. The former three classifications were grouped as non-neoplasia polyps (NNP), and the last three were defined as advanced or neoplastic polyps (NP). Clinicopathological factors, including age, sex, and the anatomic subsite of lesions in the intestinal lumen, were recorded at enrollment. The polyp location was classified into 3 parts: right side (tumors at the cecum, ascending colon, hepatic flexure, and transverse colon); left side (tumors at the splenic flexure, descending colon, and sigmoid colon); and the rectum. Twenty-four patients were found to have more than one polyp, and 6 patients had synchronous cancer. The tumors were surgically removed and pathologically confirmed. All patients were provided with a form of written informed consent, and the study was approved by the Institutional Review Board of Chang Gung Memorial Hospital (101-4609A3; 102-5224B).

#### Colorectal cancer patients

Fifty patients with untreated CRC were enrolled, and their fresh tumor and adjacent normal tissues had been investigated for somatic and germline mutations in our previous study [4]. The pathological classification of the tumor stage ranged from AJCC stage 0 to stage IV, with 30 early stage cases (stage 0–II) and 20 late stage cases (stage III–IV).

#### Controls

Taiwanese genome reference data were obtained from 150 healthy controls by whole genome sequencing. All controls had no family history of cancer and remained healthy at the age of 70 years old when they provide the blood samples (See Acknowledgement).

### Sample preparation and routine laboratory test

Fifty-three formalin-fixed paraffin-embedded (FFPE) polyps and 6 synchronous FFPE tumor blocks were submitted to the laboratory of anatomic pathology for inspection. One hematoxylin & eosin (H&E) slide of each sample was used for reviewing neoplastic contents and photorecorded by pathologists. The other 3–5 unstained slides of 10 mm-thick sections with cellularity larger than 20% were collected and subjected to the following DNA extraction. For the confirmation of germline mutations, EDTA blood samples were simultaneously collected from 53 patients. Genomic DNA from PBMCs were extracted using a DNeasy Blood and Tissue Kit (Qiagen) and stored at −80°C until use.

### DNA extraction procedure for FFPE sections

At least three slides of 10 μm thick FFPE tissue were added to the Covaris microTUBE (COVARIS). The samples were processed by using the Focused-ultrasonicator Covaris S220 to dissociate the paraffin and rehydrate the tissue. The extraction procedures followed the manufacturer's instructions.

### Next generation sequencing by ampliseq cancer hotspot panel

All tissue samples were subjected to the AmpliSeq Cancer Hotspot Panel Version 2 (Thermal Fisher). The panel specifically targets 50 cancer-related genes which harbors 2855 COSMIC [32] hotspots. The AmpliSeq Library was prepared according to the manufacturer's protocol. For the detailed procedures, please refer to our previous study [4].

### Germline mutation detection

Mutations found in FFPE tissue samples will be further tested by Sanger sequencing in paired blood genomic DNA to exclude germline mutations. Germline mutations are defined as a gene alteration found in both the tissue and blood DNA with an allelic frequency of approximately 50%.

### Bioinformatics analysis

Mutations located in exonic regions that were nonsynonymous changes causing amino acid alterations with allelic frequencies less than 3% in Asian populations were filtered in. To further eliminate high-artifact background from the fragmented FFPE DNA, only those mutations with a frequency of > 10% and variant coverage of > 30 were considered candidate variants for further analysis. Variants with amino acid changes were further examined for whether they were potentially damaging by using the Sorting Intolerant From Tolerant (SIFT) software, which can predict the possible impact of an amino acid substitution on the structure and function of a protein [33]. SIFT calculates the conservation value and scales the probability for each position. The SIFT score ranges from 0.0 (damaging) to 1.0 (tolerated).

### Statistical analysis

Descriptive statistics were summarized in percentages. Between-group comparisons were conducted using the chi-square test for each marker. A *P* value less than 0.05 (2-tailed) was considered statistically significant. All statistical tests were conducted using PASW Statistics 18. The protein domain structure and distribution of variants in specific proteins were plotted using DOG Version 1.0 (http://dog.biocuckoo.org).

## SUPPLEMENTARY MATERIALS FIGURES


