# Supplementary material for: Acquired somatic TP53 or PIK3CA mutations are potential predictors of when polyps evolve into colorectal cancer
Source: Oncotarget. 2017 Aug 21;8(42):72352–62. doi: 10.18632/oncotarget.20376 (PMC5641135; doi:10.18632/oncotarget.20376)
Supplement: Supplementary file 1 [file oncotarget-08-72352-s001.pdf]

Acquired somatic *TP53* or *PIK3CA* mutations are potential predictors of when polyps evolve into colorectal cancer

SUPPLEMENTARY MATERIALS

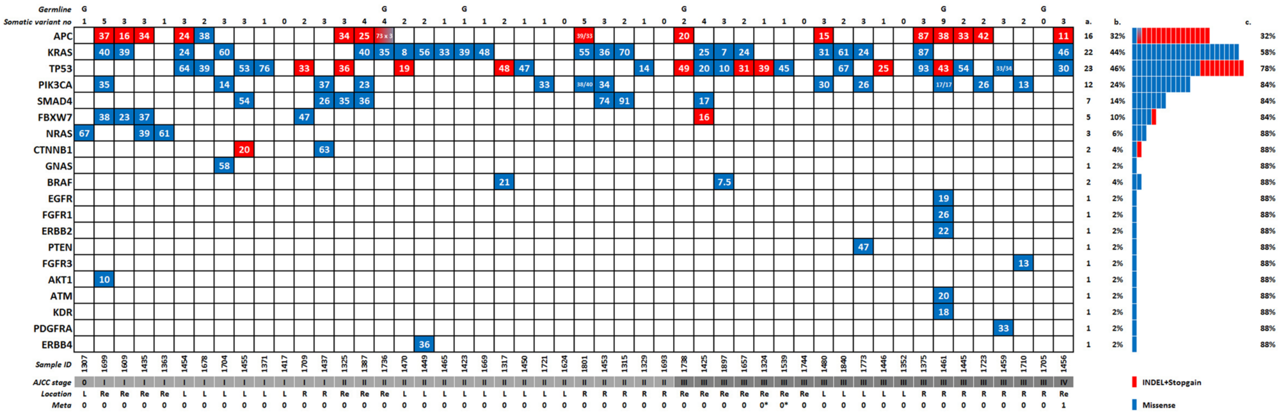

Supplementary Figure 1: Distribution of 77 somatic variants in 20 genes in the 50 CRC patients.

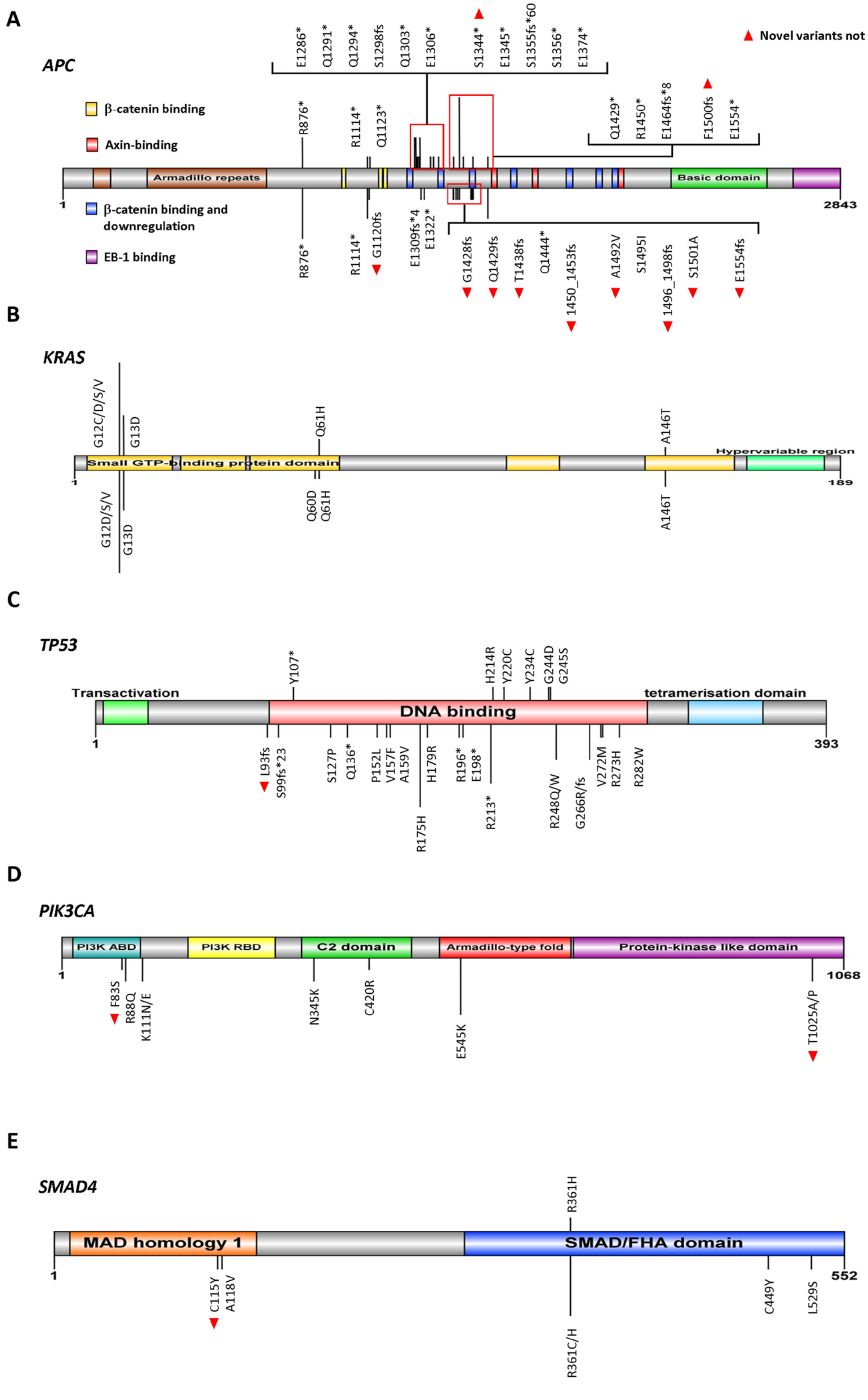

Supplementary Figure 2: Comparison of spatial distribution of somatic variants found in polyps and CRC tissues in the top 5 mutated genes. Vertical lines drawn above the protein domain denote the variants from polyps, and those below are variants from tumor tissues. The length of the line indicates the number of samples carrying the variant. A red triangle represents a novel variant that is not found in the COSMIC database. Color blocks illustrate the different functional domains of each protein. (**A**) *APC*; (**B**) *KRAS;* (**C**) *TP53*; (**D**) *PIK3CA*; (**E**) *SMAD4.*

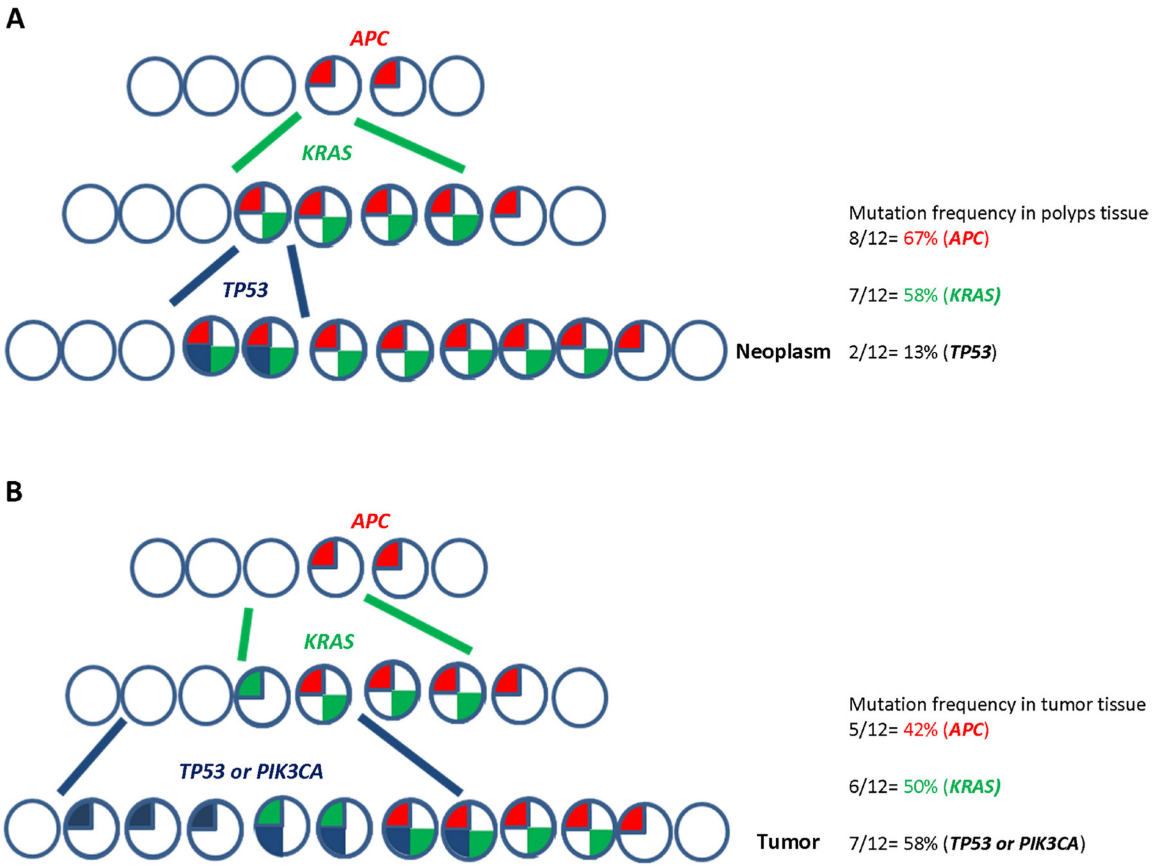

Supplementary Figure 3: Proposed clonal expansion history of adenoma (**A**) and carcinoma (**B**).
